# Co-Detection of miR-21 and TNF-α mRNA in Budding Cancer Cells in Colorectal Cancer

**DOI:** 10.3390/ijms20081907

**Published:** 2019-04-17

**Authors:** Trine Møller, Jaslin P James, Kim Holmstrøm, Flemming B Sørensen, Jan Lindebjerg, Boye S Nielsen

**Affiliations:** 1 Bioneer A/S, Hørsholm, Kogle Allé 2, 2970 Hørsholm, Denmark; trm@bioneer.dk (T.M.); jaslin.pj@gmail.com (J.P.J.); kho@bioneer.dk (K.H.); 2 Danish Colorectal Cancer Center South, Vejle Hospital, Part of Lillebaelt Hospital, Beriderbakken 4, 7100 Vejle, Denmark; flsoer@rm.dk (F.B.S.); Jan.Lindebjerg@rsyd.dk (J.L.); 3 University Institute of Pathology, Aarhus University Hospital, Palle Juul-Jensens Boulevard 99, 8200 Aarhus N, Denmark; 4 Department of Pathology, Vejle Hospital, Part of Lillebaelt Hospital, Beriderbakken 4, 7100 Vejle, Denmark; 5 Institute of Regional Health Research, University of Southern Denmark, Winsløwparken 19,3, 5000 Odense C, Denmark

**Keywords:** colorectal cancer, confocal slide scanning microscopy, inflammation, interleukin-1β, microRNA, miR-21, TNF-α, tumor budding cells

## Abstract

MicroRNA-21 (miR-21) is upregulated in many cancers including colon cancers and is a prognostic indicator of recurrence and poor prognosis. In colon cancers, miR-21 is highly expressed in stromal fibroblastic cells and more weakly in a subset of cancer cells, particularly budding cancer cells. Exploration of the expression of inflammatory markers in colon cancers revealed tumor necrosis factor alpha (TNF-α) mRNA expression at the invasive front of colon cancers. Surprisingly, a majority of the TNF-α mRNA expressing cells were found to be cancer cells and not inflammatory cells. Because miR-21 is positively involved in cell survival and TNF-α promotes necrosis, we found it interesting to analyze the presence of miR-21 in areas of TNF-α mRNA expression at the invasive front of colon cancers. For this purpose, we developed an automated procedure for the co-staining of miR-21, TNF-α mRNA and the cancer cell marker cytokeratin based on analysis of frozen colon cancer tissue samples (*n* = 4) with evident cancer cell budding. In all four cases, TNF-α mRNA was seen in a small subset of cancer cells at the invasive front. Evaluation of miR-21 and TNF-α mRNA expression was performed on digital slides obtained by confocal slide scanning microscopy. Both co-expression and lack of co-expression with miR-21 in the budding cancer cells was noted, suggesting non-correlated expression. miR-21 was more often seen in cancer cells than TNF-α mRNA. In conclusion, we report that miR-21 is not linked to expression of the pro-inflammatory cytokine TNF-α mRNA, but that miR-21 and TNF-α both take part in the cancer expansion at the invasive front of colon cancers. We hypothesize that miR-21 may protect both fibroblasts and cancer cells from cell death directed by TNF-α paracrine and autocrine activity.

## 1. Introduction

MicroRNAs are short regulatory RNAs that are formed as inactive precursors with hairpin-like structure from which a 3p and 5p strand containing 19–23 nucleotides are generated after cleavage by endonucleases [1,2,3]. The released 3p and 5p strand have different functions, but often only one of the strands associates with the RNA-induced silencing complex (RISC). The microRNA:RISC binds to unique complementary RNA sequences often located in the untranslated 3′ end of a mRNA and leads to de-stabilization or degradation of the individual target mRNA. microRNA-21 (miR-21) is one of most consistently upregulated microRNAs in cancer tissue including colorectal cancer [4,5,6,7]. miR-21 is expressed predominantly in stromal fibroblast-like cells, but is seen also in populations of cancer cells [7,8]. In colon cancer tissue, we recently described the presence of miR-21 in budding cancer cells [9]. Budding cancer cells are de-differentiated cancer cells that have detached from the cohesive, more differentiated, part of the colon cancer and are located as single cells or small clusters of cancer cells at the invasive front [10]. The prevalence of budding cancer cells in tumor microenvironment is associated with increased metastasis and poor prognosis [11,12]. The function of miR-21 in colon cancer fibroblasts and cancer cells is not clear, but its regulatory roles are likely different in different cell populations. miR-21 is involved in fibrosis as part of the transforming growth factor (TGF-β) induced fibrosis pathway [13], and targets programmed cell death-4 (PDCD4) to sustain cell survival, cancer cell invasion and metastasis [14]. miR-21 is also upregulated in inflammatory bowel disease [15,16,17], in which expression is seen in thus far uncharacterized inflammatory cells [16].

Inflammation is an inherent part of colorectal cancer progression. Several inflammatory cytokines are upregulated in cancer tissue, including tumor necrosis factor alpha (TNF-α), a potent cytokine that causes necrosis and inflammation [18,19,20] and promotes cancer [21]. Increased levels of TNF-α are associated with metastatic disease in several cancer types including colorectal cancer [22,23,24]. TNF-α is produced in cells as a type II transmembrane protein arranged in stable homo-trimers [25]. Soluble TNF-α is generated by proteolytic cleavage by TNF-α converting enzyme (TACE) that forms the active soluble homo-trimeric cytokine. TNF-α acts through TNF-α receptors (TNFR) to induce cell signaling. Interestingly, Cottonham et al. [26] showed that TNF-α in cooperation with TGF-β positively regulates the expression of miR-21 in colorectal cancer cells, and that the cells in an organoid model increase their motility and invasiveness. In addition, Qiu et al. [27] found that miR-21 expression was positively correlated with TNF-α in oral cancer cells and controlled proliferation and apoptosis, and Chen et al. [28] found that miR-21 depletion inhibited secretion of TNF-α in a rat model of diabetic nephropathy. Thus, several studies indicate an interplay between miR-21 and TNF-α putatively, of both paracrine and autocrine nature. If miR-21 regulates TNF-α protein expression, it would be expected to occur through an indirect pathway since TNF-α mRNA is not considered a direct target of miR-21 according to the microRNA target database (miRDB) (www.mirdb.org).

To study expression of individual molecules in clinical tissue samples, we have established in situ hybridization (ISH) technologies for microRNA and mRNA. Localization of the mRNA transcripts show the origin of protein synthesis in situ and thereby reveals the cellular expression in complex tissue structures. During recent years, new ISH technologies have been developed. For long RNAs, branched DNA (bDNA) probes (RNAscope) have allowed detection of mRNA expression with high specificity and high sensitivity [29]. The method allows detection of single molecules that are visible as single dots in cells and tissues and has been developed for both automated and manual procedures [30,31]. For microRNAs, the limited size of 19–23 nucleotides makes locked nucleic acid (LNA) probes advantageous [32]. LNA probes have been used for detection of multiple microRNAs both in manual and automated assays, on paraffin and frozen tissue sections, and in single-plex and du-plex applications [7,33,34,35,36].

This study was undertaken to explore expression of miR-21 and TNF-α mRNA at the invasive front of colon cancers to elucidate interplay between miR-21 and TNF-α. To do this, we developed a combined microRNA and mRNA ISH assay that allows the use of LNA probes and bDNA probes on the same section as well as enables immunohistochemical detection of cellular markers.

## 2. Results

In this study, we performed in situ expression analyses on sections from both formalin-fixed, paraffin embedded (FFPE) samples and frozen (cryo-embedded) samples. The FFPE samples were used for chromogenic single-plex analysis and the frozen samples were used for the subsequent combined multi-plex fluorescence analyses.

### 2.1. Detection of TNF-α mRNA in Colon Cancer Tissue

**Figure 1:**
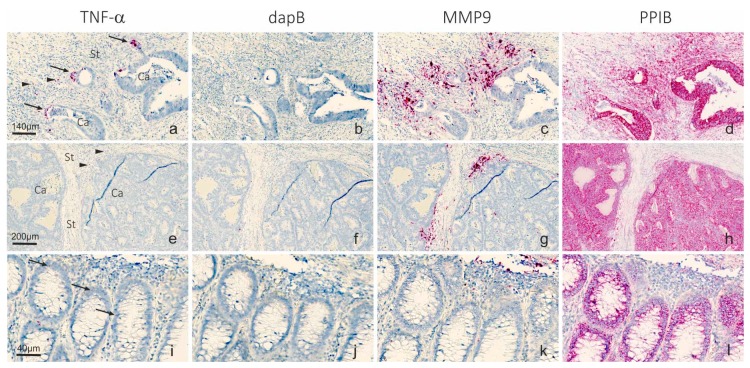
TNF-α and MMP9 mRNA in colorectal cancer samples. RNAscope probes to: TNF-α (**a**,**e**); dapB negative control (**b**,**f**); MMP9 (**c**,**g**); and PPIB positive control (**d**,**h**) mRNAs were used on serial sections from two FFPE colorectal cancer samples (**a**–**d**) with high budding and (**e**–**h**) with low budding, using automated RNAscope procedure and detection of the mRNAs with the AP and Fast Red substrate (red). In the sample shown in (**a**–**d**), an area with normal mucosa was present and expression of the RNA transcripts are shown in (**i**–**l**). Sections were counterstained with hematoxylin. TNF-α mRNA is seen as a distinct signal in a few cancer cells with branching characteristics (arrows) and a few stromal cells (arrowheads). MMP9 mRNA is very intense and seen in multiple stromal cells, typically macrophages. In normal mucosa, TNF-α mRNA is seen as single dots (presumably representing individual molecules) in a subset of the epithelial cells. The negative control probe dapB shows no signal, whereas the positive control probe to PPIB mRNA stains virtually all cells. Ca indicates cancer cell compartment; St indicates stromal cell compartment. Scale bars in (**a**–**d**): 140 µm; Scale bars in (**e**–**h**): 200 µm; Scale bars in (**i**–**l**): 40 µm.

Three FFPE samples were stained with RNAscope probes to TNF-α and matrix metalloproteinase-9 (MMP9) mRNAs together with PPIB mRNA and bacterial dapB mRNA probes as positive and negative controls, respectively (Figure 1). TNF-α mRNA ISH signal was weak and restricted to a few cells in the invasive front, where both a subset of cancer cells and stromal cells were positive. The presence of TNF-α mRNA in cancer cells was more evident in the case with high cancer cell budding (Figure 1a), whereas the two cases with low budding contained very few positive cells, and then seen in the stromal compartment (example in Figure 1e) and as occasional focal accentuation in tumor cells bordering stroma. Expression of MMP9 mRNA, which is known to be stimulated by TNF-α [37], was seen in all three samples (example in Figure 1c,g) in stromal cells with macrophage-like morphology in the expected expression pattern in the invasive front [38]. PPIB mRNA ISH signal was prominent in all three cases and stained both cancer and stromal cells, and the dapB probe resulted in no ISH signal, with only some un-specific chromogen depositions in focal areas. In one of the three cases (Figure 1a–d), a small area with normal mucosa was seen. Here, TNF-α mRNA was seen as single dots (presumably representing individual molecules) in a subset of the epithelial cells (Figure 1i–l), suggesting a very low level of expression in normal mucosa.

### 2.2. Automation of microRNA ISH, mRNA ISH and IHC in a Combined Assay

**Figure 2:**
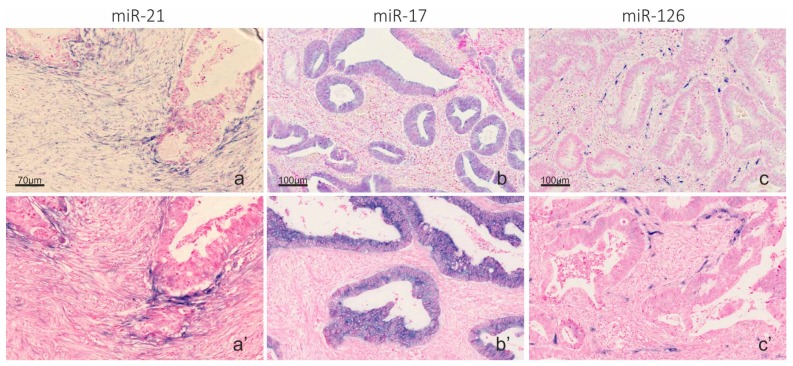
Automation of miR-21, miR-17 and miR-126 in a Ventana instrument. LNA probes to: miR-21 (**a**); miR-17 (**b**); and miR-126 (**c**) were used on FFPE colon cancer samples in a Tecan Genepaint instrument (**a**–**c**) and a Ventana Discovery Ultra instrument (**a’**–**c’**), using AP detection and NBT-BCIP substrate (blue). The sections were counterstained with nuclear fast red. At optimized experimental conditions, the Ventana and Tecan instruments showed identical staining patterns. miR-21 in stromal fibroblastic cells (**a**,**a’**); miR-17 in cancer cells (**b**,**b’**); and miR-126 in endothelial cells (**c**,**c’**). Scale bars in (**a**–**c**) are representative for (**a’**–**c’**).

**Figure 3:**
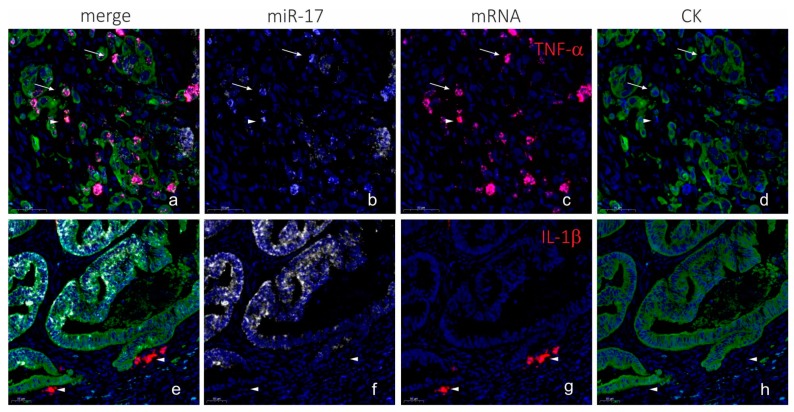
Combination of LNA ISH, RNAscope and IHC (AP procedure). Colorectal cancer sections from a cryo-embedded sample were stained for miR-17 using an LNA probe, and either TNF-α or IL-1β using RNAscope probes, and cytokeratin using immunohistochemistry. miR-17 (white in (**a**,**b**,**e**,**f**)) is seen in tumor cells in differentiated cancer cells (cohesive structures) and is absent in the de-differentiated cancer cells in areas with budding and/or branching. TNF-α mRNA (red in (**a**,**c**)) is seen in CK-positive cancer cells primarily located at the invasive front (arrows in (**a**–**d**)) and in CK-negative cells (arrowheads in (**a**–**d**)). IL-1β mRNA is seen CK-negative stromal cells (arrowheads in (**e**–**h**)). The cytokeratin staining (green in (**a**,**d**,**e**,**h**)) is seen in cancer cells including the differentiated cells of the cohesive structures and de-differentiated cells in branching and budding cancer cells. The CK staining was sub-optimal in this case—the miR-17 positive cancer cells are poorly stained with cytokeratin, and there is a dubious bleed-through of the red signal into the white channel. The blue fluorescence signal is nuclear DAPI counterstain in all images. Scale bars are indicated in lower left corner of each panel.

To combine LNA-probe based microRNA ISH and RNAscope-probe based mRNA ISH on frozen sections, we first developed the microRNA ISH assay on the Ventana instrument on FFPE sections. As test probes, we included probes to microRNAs with previously reported consistent expression patterns [33,39], including miR-126 in endothelial cells, miR-21 in stromal cells and miR-17 in cancer cells (Figure 2). The microRNA ISH assay on the Ventana instrument was found to be equally or more sensitive than the Tecan assay [7] and was found suitable for unambiguous detection of microRNAs. The microRNA staining procedure with peroxidase and TSA substrate detection [34] was then combined with RNAscope procedure, using the AP (red kit) for mRNA detection and immunohistochemistry for cell marker detection on frozen sections from a colorectal cancer. Initially, miR-17 was combined with automated TNF-α and IL-1β mRNA RNAscope probe and cytokeratin immunofluorescence (Figure 3). The combined staining revealed localization of miR-17 in the cancer cells as expected, and TNF-α mRNA was, in this case, mostly seen in CK-positive cancer cells located at the invasive front (Figure 3). In contrast, IL-1β mRNA ISH signal was limited to stromal cells in focal areas close to cancer cell de-differentiation and budding (Figure 3). miR-17 expression was lost in the cancer cells at the outer invasive front including in the budding cancer cells (Figure 3) [39].

### 2.3. Co-Localization Analyses of TNF-α and miR-21

**Figure 4:**
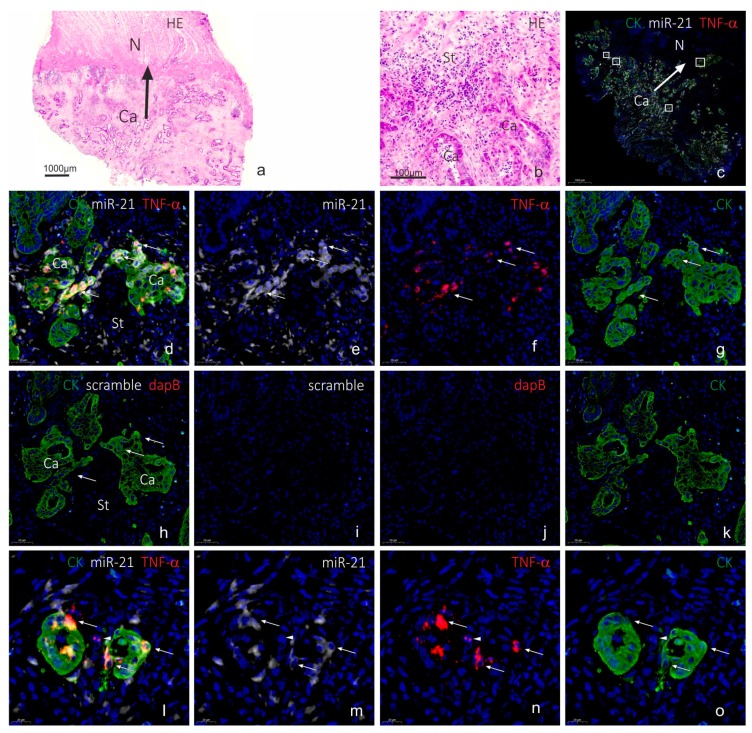
TNF-α mRNA and miR-21 in budding cancer cells in colorectal cancer (HRP procedure). The colon cancer tissue sample (cryo-embedded) is shown after H&E staining at low (**a**) and high magnification (**b**), and after combined fluorescence staining and subsequent confocal slide scanning microscopy at low magnification (**c**). The tissue section shows miR-21 (white in (**c**,**d**,**e**,**l**,**m**)) or scramble (white in (**h**,**i**)), TNF-α mRNA (red in (**c**,**d**,**f**,**l**,**n**)) or dapB mRNA (red in (**h**,**j**)) and CK (green in (**c**,**d**,**g**,**h**,**k**,**l**,**o**)). Probes to miR-21 and scramble probe were incubated on serial sections (**d**–**g**, and **h**–**k**) respectively. Arrows in (**d**–**h**) indicate the same cancer cell population in the two sections that are positive with the miR-21 and TNF-α probes and negative with the scramble and dapB probes. Arrows in (**l**–**o**) indicate miR-21 and TNF-α positive cancer cells and the arrowhead indicate a TNF-α positive stromal cell. Sections were scanned using a Pannoramic confocal slide scanner at 20× to give the overview image (**c**) and the framed areas are shown in (**d**–**g**,**l**–**o**) at high magnification (digital zoom), and two frames are shown Figure 5. Arrows in (**a**,**c**) indicate direction of invasion. Ca, cancer area; N, normal area; St, stroma area. Scale bars are indicated in lower left corner of each panel.

**Figure 5:**
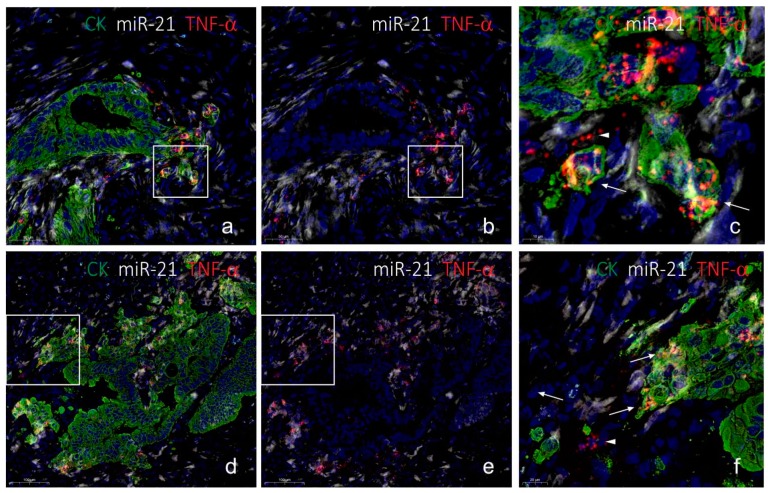
Confocal slide scanning at 40×. The same section as in Figure 4 here scanned using a 40× water objective. The two areas (**a**–**f**) show typical TNF-α mRNA expression at cancer cell branching with miR-21 expression in TNF-α-positive cells and neighboring TNF-α-positive signal in the stroma (arrowheads in (**c**,**f**)). Note that miR-21 positive stromal cells are TNF-α-negative. The RNAscope ISH signal is here evident as single dots representing individual mRNA molecules (arrows in (**c**,**f**)). Scale bars are indicated in lower left corner of each panel.

To evaluate co-localization of miR-21 and TNF-α mRNA in budding cancer cells, we performed the combined assay, detecting miR-21, TNF-α mRNA and CK, on four frozen colorectal cancer samples with evident budding characteristics. As a negative control, the scramble LNA probe was combined with dapB RNAscope and cytokeratin (CK) immunofluorescence. We changed the RNAscope detection assay to being HRP-based, using rhodamine substrate, to avoid masking effects of the Fast Red chromogen. In all four cases, TNF-α mRNA was seen in a subset of CK-positive cancer cells in the invasive front, whereas expression in (CK-negative) stromal cells was a rare event (see Figure 4). The TNF-α mRNA ISH signal was often seen in cancer cells with a branching appearance, i.e., cells that show initial outgrowth from the cohesive adenocarcinoma structure (Figure 4g,k), whereas the front-runner budding cancer cells were often TNF-α mRNA negative (Figure 4g,k). miR-21 was as expected seen in stromal fibroblastic cells, and subsets of cancer cells were also positive, including subsets of budding cancer cells (Figure 4f,j). No ISH signal was observed with the scramble probe and the dapB probe (Figure 4d), and the background fluorescence signal was limited to typical endogenous autofluorescence (Figure 4d). The combined stained slides were scanned using a confocal slide scanner with a 20× objective. Examples from the other three cases are shown in the Supplementary Materials. At 20× magnification, the TNF-α mRNA signal appeared as a rather diffuse signal. Hence, some areas of interest were scanned using a 40× water objective. At this magnification, the individual spots from the RNAscope assay were clearly visible (Figure 5).

Since expression of TNF-α mRNA was very restricted compared to miR-21, we evaluated the TNF-α positive cancer cells in the four cases for miR-21 positivity within the same cell. In two cases, virtually all TNF-α positive cells were also miR-21-positive, whereas in the other two cases only 20% or less were miR-21 positive. TNF-α positive cells located in the stroma were generally miR-21 negative. In one of the cases, we noted that a TNF-α mRNA positive cancer cell was often associated with miR-21 in neighboring cancer cells and stromal cells. However, stromal fibroblastic cells located in the vicinity of TNF-α positive cancer cells did generally not show differences in the miR-21 expression patterns compared to areas without TNF-α mRNA signal. These observations suggest that miR-21 is not co-regulated with the TNF-α mRNA, neither within the same cell nor in neighboring cells.

## 3. Discussion

This study was undertaken to address if in situ localization analyses can help to clarify interplay between miR-21 and the pro-inflammatory cytokine TNF-α during colon cancer progression. We developed a fully automated combined in situ hybridization and immunohistochemistry assay that allowed use of LNA probe technology and RNAscope technology together with immunofluorescence on the same tissue section. Using CK immunofluorescence, all cancer cells at the invasive front of the colorectal cancers were visualized. Co-localization of the TNF-α mRNA in the CK positive cells indicated that this potent pro-inflammatory cytokine is expressed in a small subset of cancer cells located at the invasive front. We found that miR-21 also co-localizes in some, but not all, of these cancer cells, suggesting that miR-21 is not co-regulated with TNF-α mRNA, and that the presence of TNF-α mRNA does not lead to suppression of miR-21 expression. In normal mucosa, TNF-α mRNA was noted as a few single dots representing very low expression and miR-21 was only occasionally seen in normal mucosa [7], indicating that the cancer or associated inflammation may be required to induce expression of the two transcripts. We speculate that miR-21 in TNF-α mRNA positive cancer cells may suppress an autocrine effect of TNF-α to mediate cell death, e.g., via PDCD4 [40].

We previously developed a microRNA ISH method on a Tecan Genepaint instrument [7], which provides highly reproducible staining results; however, we found that RNAscope was not feasible for this instrument due to reagent consumption and consequently high cost. On the other hand, fully automated RNAscope assay procedures, e.g., on a Ventana instrument [30], have been developed by the vendors (ACDbio and Roche) and implemented in our Lab. To combine microRNA ISH with RNAscope, we therefore first established a microRNA ISH procedure on the Ventana instrument. Using the AP-based staining method, and similar reagents developed previously on the Tecan instrument, we obtained identical results with a slightly improved sensitivity and signal-to-noise ratio. Because pre-treatment procedures, required for microRNA ISH and RNAscope assays, were not compatible for FFPE samples, we developed the combined assay on frozen tissue, which also has the advantage of having better RNA integrity if processed for cryo-protection immediately after resection and appropriate storage. The combination of the three techniques into a triple fluorescence assay can potentially have many applications, including co-localization studies of a microRNA and its target mRNA and protein.

We investigated four cases of colorectal cancer that all included the invasive tumor front and prevalent cancer cell budding. In each of the four cases, multiple events of TNF-α mRNA expression and miR-21 expression in cancer cells could be evaluated. Because the same localization pattern was noted for TNF-α mRNA in the four cases, and also that the four cases showed the similar miR-21 localization pattern, we assume that our findings are representative for such adenocarcinomas despite the limited number of cases. The invasive growth pattern, however, varied with respect to the cancer cell structures, the presence of inflammation and fibrosis. In all four cases, the localization of TNF-α mRNA was similar, thus TNF-α positive cancer cells were often located at branching points of the cohesive adenocarcinoma structures located toward the invasive front or in detached de-differentiated budding cancer cells or in larger islands of cancer cells. TNF-α mRNA positive cancer cells were only occasionally seen in the central areas of the tumors. The characteristic TNF-α mRNA expression pattern was seen in both paraffin and cryo-embedded samples, and the same pattern was obtained with both the AP- and HRP-based RNAscope kits. Differences in staining intensity and prevalence of positive cells may be related to biological differences or differences in mRNA integrity. For specificity evaluation of the RNAscope assay, we included reference probes, of which, most importantly, the negative control probe resulted in the lack of ISH signal except from background. RNAscope probes to mRNAs for MMP9, PPIB and IL-1β all showed the expected expression patterns. Taken together, the observations suggest that the ISH signal obtained with the TNF-α probe is representing the genuine TNF-α mRNA. To our knowledge, this is the first study to show expression of TNF-α mRNA in budding and branching colon cancer cells in situ. The particular localization of the TNF-α mRNA expressing cells suggests a role in aggressive cancer cell invasion. In support of this observation, Li et al. [41] measured increased TNF-α mRNA levels in total RNA isolated from micro-dissected colorectal cancer budding cells and the surrounding microenvironment. In addition, TNF-α has been found to be involved in branching morphogenesis in in vivo models, e.g., during rat mammary gland development facilitated by MMP9 [42,43], suggesting that the cancer cells re-establish cellular mechanism used during early organ development. It is also of particular interest that TNF-α was found to induce an invasive phenotype in ovarian epithelial cystic structures [44]. In the latter study, TNF-α was added to the growth medium with the cystic structures, suggesting a paracrine mechanism inducing invasion, which would be expected if the source of TNF-α was inflammatory cells, including TNF-α-loaded neutrophilic granulocytes that may not carry the TNF-α mRNA. The observations in our study suggest that TNF-α also mediate an autocrine mechanism that contributes to cancer invasion. A larger sample set would be required to address if the expression of TNF-α mRNA by ISH or the co-expression with miR-21 is of prognostic relevance.

Digital slides obtained by confocal slide scanning microscopy allowed systematic evaluation of individual cells. We evaluated TNF-α mRNA and CK positive cells in each of the four cases for the presence of miR-21. The miR-21 positive fraction varied from 20% to 100%, suggesting no general correlation. In one of the cases, we noted that a TNF-α mRNA positive cancer cell was often associated with miR-21 in neighboring cancer cells and stromal cells. This observation could suggest that TNF-α stimulates miR-21 in various cells in the local neighborhood independent of the cell type. TNF-α has been reported to upregulate miR-21 expression in a variety of cell types, including human renal epithelial cells [45] and in Caco-2 cells used in an intestinal barrier model [46]. In a study by Xu et al. [47], it was observed that the expression of miR-21 increased gradually with low concentrations of TNF-α, while being suppressed at high concentrations. Thus, TNF-α is likely to have an effect on miR-21 expression, but in a complex tissue with many different cells and different signaling pathways interacting, distinct correlation patterns can be difficult to decipher. This may explain why no obvious correlation between TNF-α mRNA in cancer cells and stromal miR-21 was observed. Such patterns, may be better characterized in early stage cancers or in appropriate in vitro cell models. Conversely, miR-21 may control TNF-α expression. Ando et al. [48] found that a miR-21 mimic upregulated TNF-α in T-cells and Zhang et al. [49] reported that miR-21 directly targets TNF-α in bronchial epithelial cells. Future studies may disclose whether miR-21 and TNF-α are cooperating, competing or completely independent during cancer progression.

In conclusion, we report that miR-21 and TNF-α mRNA both are expressed at the invasive front of colon cancers and are co-localized in a subset of budding cancer cells and cells located at branching points and in clusters of cancer cells. Our attempts to clarify potential interactions between miR-21 and TNF-α did not result in consistent co-expression or converse expression patterns. If miR-21 expression is suppressed by TNF-α, therapeutic use of Infliximab would increase miR-21, which would promote tumor progression. Therefore, better understanding of the TNF-α/miR-21 interplay is highly warranted.

## 4. Material and Methods

### 4.1. Tissue Material

Three FFPE and four frozen colon adenocarcinoma tissue samples (Table 1) were obtained from Asterand (BioIVT, W Sussex, UK). The tissue samples were selected according to resection year (2015–2017), a well-defined invasive area and, for the frozen samples, also evident tumor cell budding and high RNA integrity number (RIN) values (>7). Evaluation of tissue sections stained with hematoxylin and eosin showed that all cases were moderately differentiated adenocarcinomas. The Asterand/BioIVT tissue samples were obtained according to the ethical principles defined in the Nuremburg Code, on the recommendations to consider when sourcing human biospecimens (https://info.bioivt.com/biospecimen-sourcing-white-paper).

### 4.2. Probes for in situ Hybridization

Double-labeled locked nucleic acid (LNA™) probes were obtained from Qiagen (Exiqon-Qiagen, Hilden, Germany) as double digoxigenin-labeled LNA:DNA chimeric oligos. The microRNA antisense probes had approximately 30% of DNA replaced by LNA to increase the binding affinity (RNA T_m_): miR-21-5p (TCAACATCAGTCTGATAAGCTA, RNA T_m_ = 83 °C), miR-17-5p (TACCTGCACTGTAAGCACTTT, RNA T_m_ = 89 °C), miR-126-3p (CATTATTACTCACGGTACGA, RNA T_m_ = 84 °C), and scramble (ATGTAACACGTCTATACGCCCA, RNA T_m_ = 86 °C). The microRNA probes recognize both the mature forms as well as precursor forms. RNAscope probes, or branched DNA (bDNA) probes, are designed to have high specificity and high sensitivity (www.acdbio.com). The high specificity is obtained by design of two antisense DNA oligonucleotides, also called double-Z probes, to bind adjacent sequences as pairs on the target sequence. The high sensitivity is partly based on design of typically up to 20 pairs for individual mRNA targets. The following RNAscope probes were obtained from ACD, Biotechne (Newark, CA): TNF-α (Tumor necrosis factor, target region: 67–1079, 13 zz pairs), IL-1β (Interleukin-1, target region: 2–1319, 20 zz pairs), MMP9 (matrix metalloproteinase-9, target region: 93–1422, 30 zz pairs), dapB (a *Bacillus subtilis* gene, 414–862, 10 zz pairs), and PPIB (Cyclophilin B, 139–989, 16 zz pairs).

### 4.3. Automated Chromogenic LNA ISH

MicroRNA ISH was performed using LNA probes on 5-µm-thick FFPE sections essentially as described previously [7], in which the specificity of the ISH staining was also analyzed. In brief, sections were deparaffinized and proteinase-K treated. Probes were added to the sections and incubated at concentrations and hybridization temperatures described earlier [7,39]. The probes were detected with alkaline phosphatase (AP) conjugated anti-DIG antibodies and stained with NBT-BCIP substrate for 30–60 min. In the Ventana instrument (Roche, Basel, Switzerland), protease-1 was used for tissue pre-treatment and probes were incubated at 2nM (miR-21) or 10nM (miR-17 and miR-126).

### 4.4. Automated Chromogenic RNAscope

RNAscope ISH was performed on 5-µm-thick FFPE sections essentially as described by Anderson et al. [30] using the AP (red) kit at recommended experimental conditions for the Ventana Discovery Ultra instrument. In this setup, we used Amp5 for 32 min (MMP9, IL-1β) or 60 min (TNF-α, dapB, PPIB).

### 4.5. Automated Combined ISH and IHC

Ten-micrometer-thick sections were obtained and fixed overnight in 4% paraformaldehyde. Staining of microRNA in frozen tissue sections has previously been described [35], using fluorescence detection with horse-radish-peroxidase (HRP)-conjugated anti-DIG antibodies and TSA-Cy5 substrate [34] (Roche). RNAscope ISH was performed as recommended by the manufacturer using the AP kit (Fast red substrate, Figure 3) or the HRP kit (TSA-rhodamine substrate, Figure 4, Figure 5 and Figure S1) in a Ventana Discovery Ultra instrument (Roche) [30]. Blocking of HRP in between the LNA assay and the RNAscope assay was done using the Discovery inhibitor for 10 min (Ventana, Roche Diagnostics). For cytokeratin immunofluorescence, the AE1/3 mouse monoclonal antibody (Dako-Agilent, Glostrup, Denmark) was used at 1:500 and detected with Alexa-488 conjugated anti-mouse Ig (Jackson Immunoresearch, West Grove, PA, USA). The negative control slides used as reference included replacement of the miR-21 probe with the scramble probe and replacement of the TNF-α probe with the dapB probe, whereas the anti-cytokeratin was retained, and was performed on cryo-samples A, B and C. Here, the miR-21 probe was incubated at 10 and 20 nM and the scramble probe at 20 nM. Despite a difference in staining intensity, there was no difference in the cellular staining pattern of the miR-21 signal when comparing the miR-21 probe concentration of 10 and 20 nM. In Figure 4, Figure 5 and Figure S1, the images were acquired from slides incubated with 20 nM probe concentration equal to the scramble probe. Sections were mounted with a DAPI-containing anti-fade solution, ProLong Gold (Thermo Fisher Scientific, Waltham, MA, USA). The detailed procedure is proprietary of Bioneer A/S (Bioneer, Hørsholm, Denmark).

### 4.6. Slide Scanning and IMAGE Acquisition

All chromogen stained slides were scanned on a Zeiss Axioscan equipped with a 20× objective. All images in Figure 1 and Figure 2 were obtained from such digital slides. Confocal slide scanning was performed using a Pannoramic confocal scanner (3DHISTECH Ltd., Budapest, Hungary), which provides confocal images made by LED light and structured illumination technology. The use of this slide scanner has previously been described in detail for use on sections stained for miR-21 and two subsequent immunoperoxidase stainings [9]. The scanner was equipped with a 20× objective (NA = 0.8, Zeiss, Oberkochen, Germany) and a 40× water immersion objective (NA = 1.2, C-Apochromat (W), Zeiss, Oberkochen, Germany). For the current study, the following LED light sources were applied in the excitations: DAPI 390/22 nms, 520 mW, Cy3 555/28 nms, 370 mW, Cy5 635/22 nms, 510 mW, FITC 475/28, 530 mW. We used one dual pass filter (for FITC and Cy5, custom designed by 3D HisTech) and two single pass filters (TRITC/rhodamine and DAPI, both Semrock, New York, NY, USA). Digital slides were obtained with manually adjusted settings with regards to exposure time, digital gain and excitation intensity. The image acquisition settings for all 4 fluorophores were set at full excitation intensity if not otherwise stated. Image acquisition parameters were: miR-21 (Cy5, exposure time varying from 20–40 ms with digital gain 1), TNF-α mRNA (rhodamine, 20 ms, with 15–30% of full excitation intensity), CK immunofluorescence (FITC, 74–174 ms, digital gain 2), and nuclear counterstain (DAPI, 176–325 ms, digital gain 2). The settings applied to the individual cases were also used on the respective negative control sections incubated with scramble LNA probe and dapB mRNA RNAscope probe (cryo samples A, B, and C). Seven confocal layers of 1 µm were obtained and assembled into one extended focus layer, from which we obtained all fluorescence images in this paper.

## 5. Conclusions

In this study we developed a combined microRNA and mRNA ISH assay that allows the use of LNA probes and RNAscope probes on the same section as well as enables immunohistochemical detection of cellular markers. We explored expression of miR-21 and TNF-α mRNA at the invasive front of four colon cancers in order to elucidate expression interplay between miR-21 and TNF-α, and found that they are both expressed at the invasive front of colon cancers and are often co-detected in budding cancer cells and cancer cells considered to be part of branching events, however, the co-localization analysis of the four cases did not show consistent co-expression or converse expression patterns.

## Figures and Tables

**Table 1:** Tissue samples.

Case	Procurement	Type	Differentiation	Budding	ISH Method
1	FFPE-A	Ad	Mod	1 (BD1)	Chromogen
2	FFPE-B	Ad	Mod	2 (BD1)	Chromogen
3	FFPE-C	Ad	Mod	10 (BD3)	Chromogen
4	Cryo-A	Ad	Mod	12 (BD3)	Fluorescence
5	Cryo-B	Ad	Mod	6 (BD2)	Fluorescence
6	Cryo-C	Ad	Mod	6 (BD2)	Fluorescence
7	Cryo-D	Ad	Mod	5 (BD2)	Fluorescence

Ad, adenocarcinoma; BD, budding density (according to guidelines issued by International Tumor Budding Consensus Conference, April 2016); FFPE, formaldehyde fixed, paraffin embedded; Mod, moderately differentiated. Budding scores: BD1: 0–4, BD2: 5–9, BD3: >10 buds per 0.785 mm^2^ field of view.

## Supplementary Materials

Supplementary materials can be found at https://www.mdpi.com/1422-0067/20/8/1907/s1. Figure S1. Confocal slide scanning of sections submitted to automated combined staining of miR-21, TNF-α mRNA and CK. The three panels in this supplementary figure show miR-21 and TNF-α mRNA expression in 3 different colorectal cancer cases with varying invasion patterns (cases cryo-B, cryo-C and cryo-D, Table 1). Examples are shown at low (a–d) and high (e–h) magnification, and in a, the framed area is depicted in e–h. In example cryo-B, TNF-α mRNA is seen in foci of cancer cells also positive for miR-21. In example cryo-C, TNF-α mRNA is prevalent and seen in multiple cancer cells that are generally weakly stained for miR-21 or miR-21 negative, whereas miR-21 is prevalent in the stromal cells. In example cryo-D, miR-21 is seen in a few TNF-α mRNA expressing branching cancer cells. For Cryo-B and Cryo-C, in a serial tissue section, the miR-21 probe was replaced with an LNA scramble probe, and the TNF-α mRNA probe was replaced with the dapB mRNA RNAscope probe, that both show virtually no staining (magnification in e–h is identical to i–l).
