# Supplementary material for: Co-Detection of miR-21 and TNF-α mRNA in Budding Cancer Cells in Colorectal Cancer
Source: Int J Mol Sci. 2019 Apr 17;20(8):1907. doi: 10.3390/ijms20081907 (PMC6515373; doi:10.3390/ijms20081907)
Supplement: Supplementary file 1 [file ijms-20-01907-s001.pdf]

Cryo-B

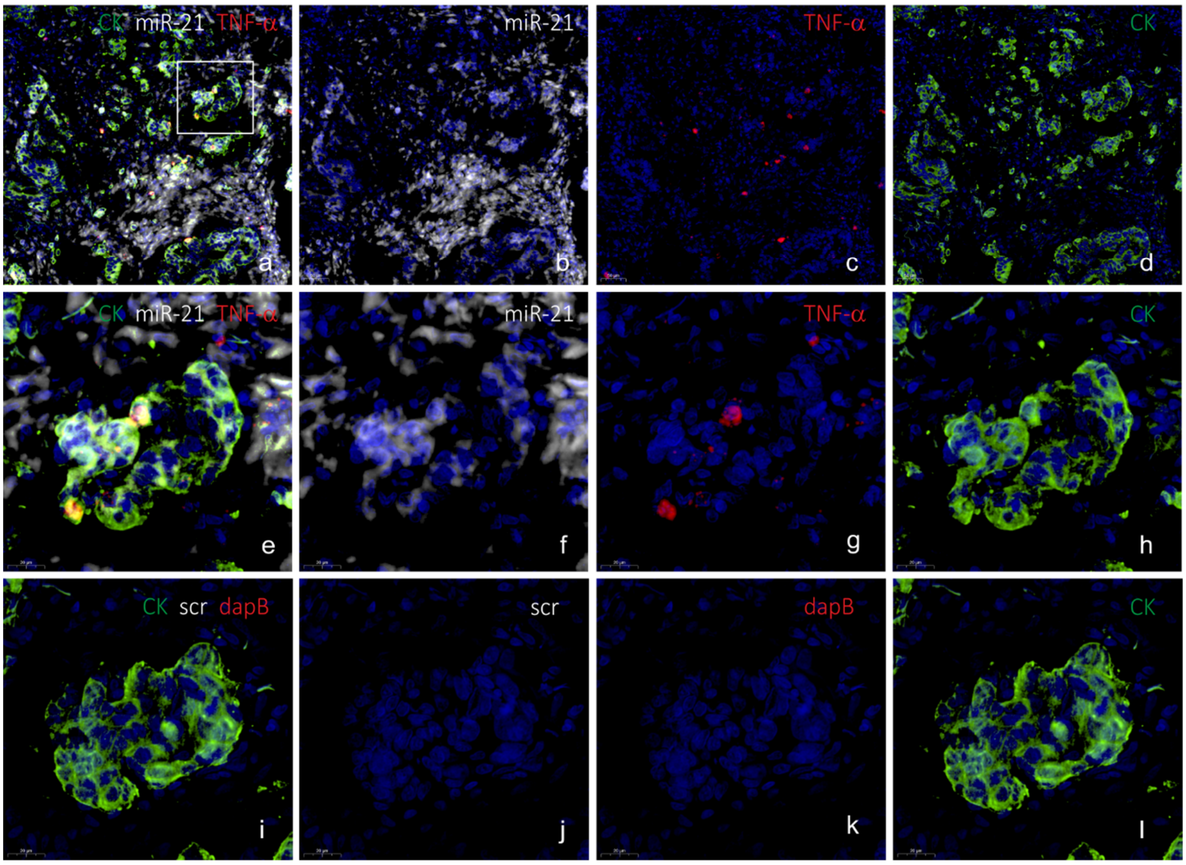
CK miR-21 TNF-α
miR-21
TNF-α
CK
a
b
c
d
CK miR-21 TNF-α
miR-21
TNF-α
CK
e
f
g
h
CK scr dapB
scr
dapB
CK
i
j
k
l

Cryo-C

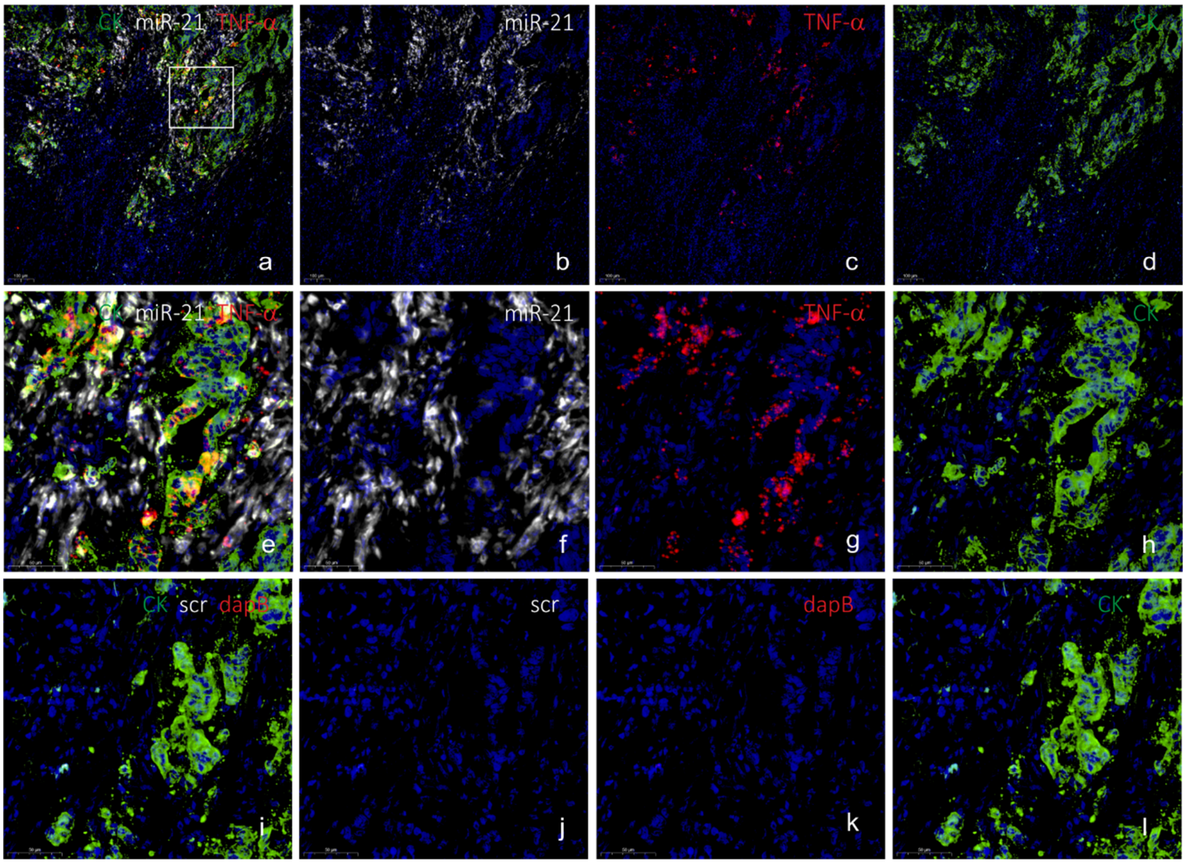
CK miR-21 TNF-α
miR-21
TNF-α
CK
a
b
c
d
CK miR-21 TNF-α
miR-21
TNF-α
CK
e
f
g
h
CK scr dapB
scr
dapB
CK
i
j
k
l

Cryo-D

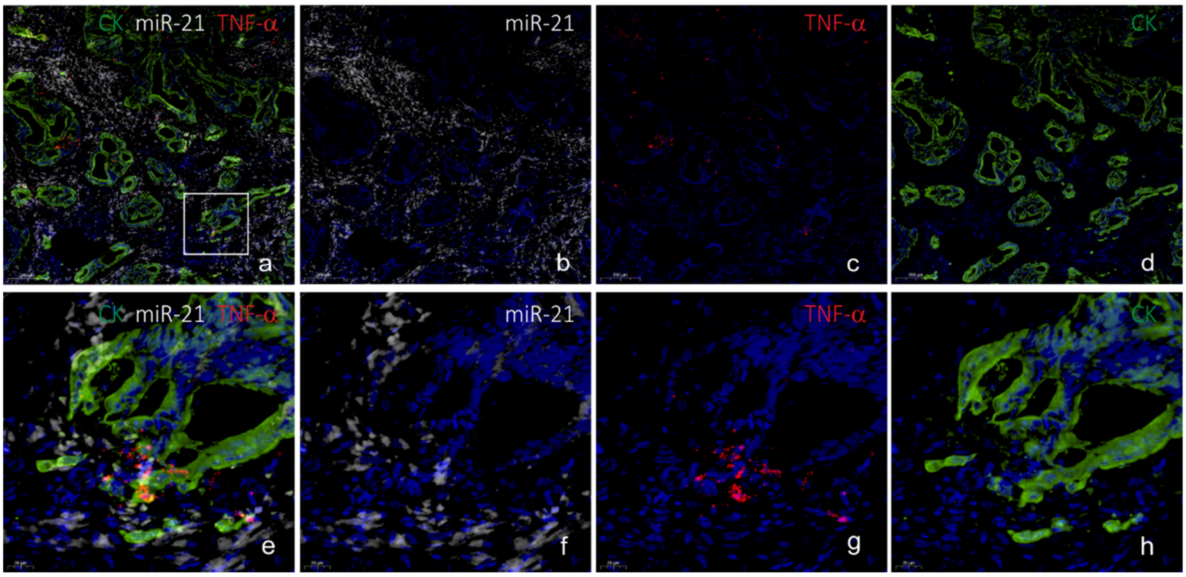

Figure S1. Confocal slide scanning of sections submitted to automated combined staining of miR-21, TNF-α mRNA and CK. The three panels in this supplementary figure show miR-21 and TNF-α mRNA expression in 3 different colorectal cancer cases with varying invasion patterns (cases cryo-B, cryo-C and cryo-D, Table 1). Examples are shown at low (a–d) and high (e–h) magnification, and in a, the framed area is depicted in e–h. In example cryo-B, TNF-α mRNA is seen in foci of cancer cells also positive for miR-21. In example cryo-C, TNF-α mRNA is prevalent and seen in multiple cancer cells that are generally weakly stained for miR-21 or miR-21 negative, whereas miR-21 is prevalent in the stromal cells. In example cryo-D, miR-21 is seen in a few TNF-α mRNA expressing branching cancer cells. For Cryo-B and Cryo-C, in a serial tissue section, the miR-21 probe was replaced with an LNA scramble probe, and the TNF-α mRNA probe was replaced with the dapB mRNA RNAscope probe, that both show virtually no staining (magnification in e–h is identical to i–l).
